# A Computationally Efficient Approach to Segmentation of the Aorta and Coronary Arteries Using Deep Learning

**DOI:** 10.1109/ACCESS.2021.3099030

**Published:** 2021-07-21

**Authors:** Wing Keung Cheung, Robert Bell, Arjun Nair, Leon J. Menezes, Riyaz Patel, Simon Wan, Kacy Chou, Jiahang Chen, Ryo Torii, Rhodri H. Davies, James C. Moon, Daniel C. Alexander, Joseph Jacob

**Affiliations:** 1 Centre for Medical Image ComputingUniversity College London 4919 London WC1V 6LJ U.K.; 2 Department of Computer ScienceUniversity College London 4919 London WC1V 6LJ U.K.; 3 Hatter Cardiovascular Institute, University College London 4919 London WC1V 6LJ U.K.; 4 Department of RadiologyUniversity College London Hospital London NW1 2BU U.K.; 5 Institute of Nuclear Medicine, University College London 4919 London WC1V 6LJ U.K.; 6 Institute of Cardiovascular Science, University College London 4919 London WC1V 6LJ U.K.; 7 Department of Mechanical EngineeringUniversity College London 4919 London WC1E 7JE U.K.; 8 Barts Heart Centre London EC1A 7BE U.K.; 9 Department of Respiratory MedicineUniversity College London 4919 London WC1V 6LJ U.K.

**Keywords:** Aorta, computed tomography coronary angiography, coronary artery, deep learning, segmentation

## Abstract

Early detection and diagnosis of coronary artery disease could reduce the risk of developing a heart attack. The coronary arteries are optimally visualised using computed tomography coronary angiography (CTCA) imaging. These images are reviewed by specialist radiologists who evaluate the coronary arteries for potential narrowing. A lack of radiologists in the UK is a constraint to timely diagnosis of coronary artery disease, particularly in the acute accident and emergency department setting. The development of automated methods by which coronary artery narrowing can be identified rapidly and accurately are therefore timely. Such complex computer based tools also need to be sufficiently computationally efficient that they can run on servers typically found in hospital settings, where graphical processing units for example are unavailable. We propose a fully automatic two-dimensional Unet model to segment the aorta and coronary arteries on CTCA images. Two models are trained to segment two regions of interest, (1) the aorta and the coronary arteries or (2) the coronary arteries alone. Our method achieves 91.20% and 88.80% dice similarity coefficient accuracy on regions of interest 1 and 2 respectively. Compared with a semi-automatic segmentation method, our model performs better when segmenting the coronary arteries alone. The performance of the proposed method is comparable to existing published two-dimensional or three-dimensional deep learning models. Importantly, the algorithmic and graphical processing unit memory efficiencies are maintained such that the model can be deployed without requiring graphical processing units, and therefore can be used in a hospital setting.

## I. Introduction

Coronary artery disease (CAD) is one of the leading causes of death in the UK [1] and worldwide. The lumen of the coronary arteries can narrow as a result of build-up of atheromatous plaque within the artery wall. Reductions in local blood flow as a result of vessel lumen narrowing can starve the heart muscle of oxygen. Vulnerable plaques can rupture, occlude the vessel lumen and result in cardiac muscle ischaemia/death, which manifests clinically as a heart attack. Early detection of the presence of atheromatous plaque and vessel stenosis [2] could allow early medical intervention and potentially reduce the risk of heart attack. Currently, several non-invasive imaging modalities are available to clinicians for visualising the anatomy of the coronary arteries as well as delineating the severity of vessel stenosis. These imaging modalities are Stress Echocardiography [3], Cardiac magnetic resonance imaging (MRI) [4] and Computed Tomography Coronary Angiography (CTCA) [5]. CTCA is the quickest of these methods and offers high sensitivity and specificity for detection and exclusion of significant coronary stenosis [6]. As a result CTCA is the preferred first-line option for the assessment of stable cardiac disease in the National Institute for Health and Care Excellence guidelines for the UK [7]. CTCA has the ability to identify calcified, non-calcified coronary plaque and mixed-attenuation plaques which can help clinicians characterise plaques and formulate management strategies.

To assess the severity of CAD, one approach involves visual estimation of stenosis severity on CTCA scans. This requires the geometrical information of the coronary arteries to be provided in order to accurately assess the severity of the stenosis. This approach however is subjective, time consuming and requires specialist radiologists, who are in short supply, to interpret the CTCA images [8]. Accurate interpretation and diagnosis of CAD is heavily reliant on the experience and expertise of individual clinicians [9], [10]. The diagnostic outcomes can differ between newly trained clinicians compared to experienced specialists.

An alternative approach for assessing CAD severity involves performing computational fluid dynamics (CFD) on the target arteries [11]. It first requires identification of accurate geometries of the aorta and coronary arteries. A set of partial differential equations of blood flow are then solved numerically given the boundary conditions and the geometries. Once blood flow is estimated, the useful clinical predictor, Fractional Flow Reserve (FFR) [12]–[14] can be derived. Provided accurate geometries of the aorta and coronary arteries are available, this approach provides an objective estimation of stenosis and does not require additional imaging, which makes it particularly attractive to clinicians.

Deriving accurate geometrical information of the aorta and coronary arteries is important for the above approaches. It is achieved by delineating the outline of the vessels, termed segmentation. Blood vessel segmentation can be performed manually, semi-automatically or automatically [15]–[20]. Manual segmentation is subjective and time consuming, requiring pixel-by-pixel labelling of individual vessels. Semi-automatic and automatic segmentation methods are objective and quicker, though they can require manual correction for under- or over- segmented vessels. There remains an unmet need to develop fast, objective and accurate automated computer-derived coronary artery segmentation algorithms that can be deployed in a hospital setting to assist clinicians diagnose CAD. This is especially relevant in Accident and Emergency (A+E) departments where CTCA reviews are often delayed due to a lack of available specialists to read the CTCAs [21]. This delay in turn slows patient management in A+E, increases resource utilisation and results in excess costs to the health service.

Deriving accurate geometries of the aorta and coronary arteries are crucial components of the clinical information required by radiologists and cardiologists to evaluate the severity of coronary artery disease. The current approaches used to segment the coronary arteries and the aorta have varying accuracy, can be time consuming and most importantly require extensive computational resources. Most hospital systems in the UK do not have access to the required computational resources such as GPUs required for machine learning analyses. We have proposed a deep learning approach to improve the accuracy and speed of cardiac vessel analysis. Our proposed approach is fast, accurate and fully automatic. But most importantly, our method requires only low computational resources, which are feasible to be implemented within a hospital network.

A fully automatic detection and classification system for CAD using computer-based deep learning algorithms is a way to achieve the above goal. The first stage of the process initially requires segmentation (identifying the outlines) of the aorta and coronary arteries on CTCA images. The second stage involves classification of disease severity performed on segmented CTCA images. The segmentation task has be considered in two ways in the literature. The first involves segmentation of both the aorta and the coronary arteries. For example, Gu *et al.* [22] proposed a 3D deep learning model to perform this task. The other strategy is to segment just the coronary arteries alone. For example, Huang *et al.* [23] suggested a 3D deep learning method with centreline to segment the coronary arteries. In general, the performance of aorta and coronary artery segmentation is better than segmentation of the coronary arteries alone. A more detailed summary regarding existing deep learning-based segmentations is discussed in the following paragraph.

The utility of deep learning applied to medical images [24] and relevant work in medical image segmentation have been extensively discussed in the literature [25]–[27]. The works related to artery segmentation on CTCA images have been discussed in two review papers [20], [28]. They provide a review of 3D vessel lumen segmentation techniques and deep learning methods for cardiac image segmentation. The focus of the current study relates to deep learning methods for coronary artery segmentation and we briefly summarise the work published in the following paragraph.

Several deep learning techniques have been proposed to segment the aorta and/or coronary arteries. Moeskops *et al.* [29] investigated a single convolutional neural network trained to segment coronary arteries in cardiac CTA images. The training dice similarity coefficient (DSC) accuracy was around 65%. A 3D-convolutional neural network was presented by Merkow *et al.* [30], which demonstrated that processing the volumetric data in 3D could improve the segmentation performance compared to 2D processing. However, the performance of the coronary artery segmentation model was not reported. Kjerland [31] adopted a 3D DeepMedic network to segment both the aorta and coronary arteries. The reported DSC accuracy was between 75%–78%. Huang *et al.* [23] examined a 3D Unet with/without a centerline to segment the coronary artery. The DSC accuracy was between 71%–78%.

Recently, a 3D multi-channel Unet has been proposed by Chen *et al.* [32], which had a DSC accuracy of 80% for coronary artery segmentation. Shen *et al.* [33] proposed a 3D fully convolutional network with attention gates to segment both the aorta and coronary artery. The boundary of the segmented artery was smoothed by a level set function. The average DSC accuracy was about 90%. Lee *et al.* [34] introduced a template transformer network where a shape template is deformed to match the underlying structure of interest through an end-to-end trained spatial transformer network for coronary artery segmentation. The DSC accuracy is between 76%–78%. Wolterink *et al.* [35] proposed using graph convolutional networks to predict the spatial location of vertices in a tubular surface mesh that segments the coronary artery lumen. The average DSC is 74%. Mirunalini *et al.* [36] proposed a two-stage approach to segment the coronary artery. The first stage adopted a 2D Recurrent Convolutional Neural Network to detect the artery in the slice, then a 2D residual Unet was used to segment the coronary artery. The intersection over union (IoU) was reported, which was 84%. Lei *et al.* [37] developed a 3D Attention Fully Convolutional Network model to automatically segment the aorta and coronary artery for CCTA. The mean DSC is 83%. Gu *et al.* [22] recently published a 3D global feature embedded network with active contour loss to segment the aorta and coronary artery. The reported average DSC is 91.43%.

Recently, a multi-objective clustering and toroidal model-guided tracking method has been used to segment the coronary artery tree automatically [38]. Gao *et al.* [39] have proposed a novel deep neural network solution (TreeVes-Net) that allows machines to perceive FFR values directly from static coronary CT angiography images.

There is a lack of efficient deep learning methods (which only require low computational resources) that can be used within typical hospital networks. For example, 3D deep learning models require a high computational resource. Though a 2D deep learning method has been described in the literature [36], the second additional component that was part of this model would increase the computational complexity of the model and hence decrease the algorithmic efficiency of the method. We propose a 2D Unet to perform aorta and/or coronary artery segmentation and demonstrate that this 2D Unet is practically feasible to be implemented given that the computational resources are limited to those available in a hospital network whilst maintaining a good segmentation accuracy. Additionally, we emphasise that we are the first to employ a 2D technique (2D Unet) for artery segmentation directly on the CTCA image. Most of the previous studies evaluating coronary artery segmentation have utilised 3D models. For the study that employed a 2D approach, where an additional sequence model was required for segmentation, the coronary artery segmentation was performed indirectly.

In this study, we propose a modified U-Net [40] model: (1) a batch normalization layer is added to the convolution block; (2) a dropout layer is added before each convolution block and evaluate its performance for the automated segmentation of the aorta and coronary arteries on CTCA images. We then retrain our model to segment the coronary arteries alone and demonstrate improved performance.

The main contributions of this study are:

• The first study to propose a modified 2D Unet that directly segments the aorta and/or coronary arteries on CTCA scans
• The method is practically feasible to be implemented within clinical systems where the available computational resources are limited
• Importantly, our technique works well when the coronary arteries alone are segmented (accuracy ~89%).

## II. Method

All the patients in the study had presented with chest pain and associated symptoms that indicated an intermediate risk of coronary artery disease. All patients underwent cardiac CT angiography for anatomical assessment of their coronary arteries and risk stratification for coronary artery disease. 71 cases were selected out of a total of 101 cases. 30 cases were not usable mainly due to major motion artefacts, artefacts on the image or the presence of coronary stents or bypass grafts distorting the coronary anatomy. The Simpleware ScanIP algorithm also failed in vessel segmentation on two of the selected cases. To segment the aorta and/or coronary artery we have proposed a modified 2D Unet model where: (1) a batch normalization layer has been added to the convolution block; (2) a dropout layer is added before each convolution block. The proposed method is compared to semi-automatic segmentation (Simpleware-ScanIP) and 2D/3D deep learning methods. The segmentation performance and time taken for vessel analysis are then evaluated.

### A. Clinical Data

**FIGURE 1.**
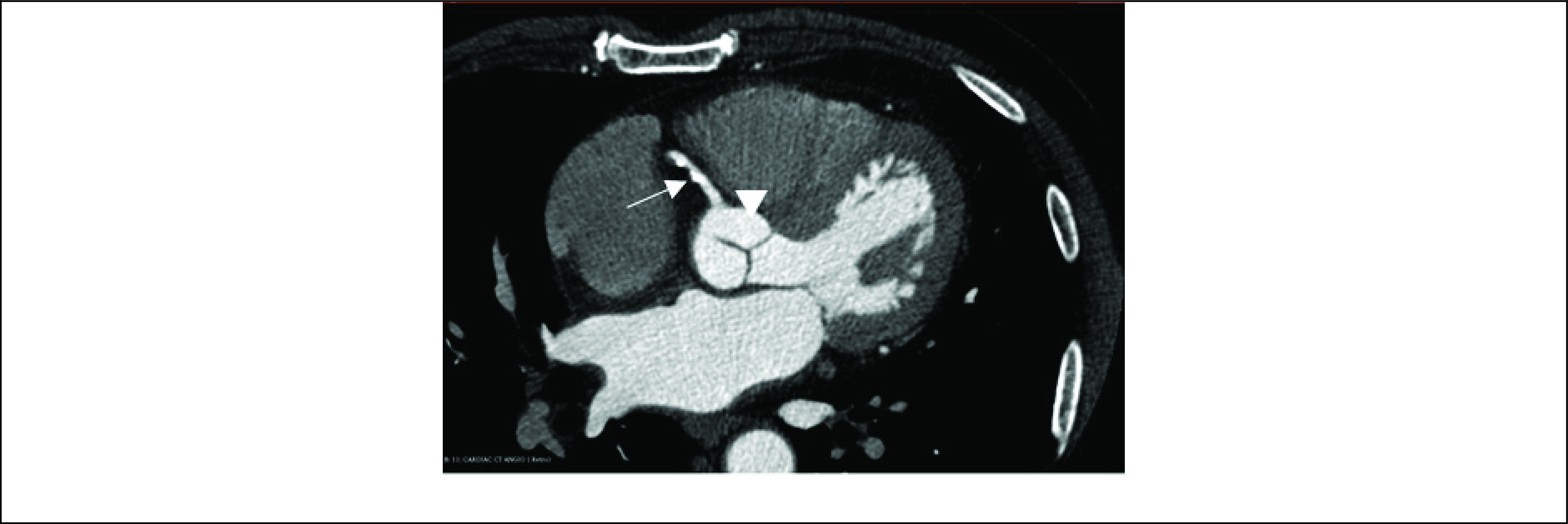
A CTCA scan of a patient. Arrow = right coronary artery; arrowhead = ascending aorta.

The final study data represented CTCA scans performed on 69 subjects with chest pain. The scans were acquired at University College Hospital London and Barts Health NHS Trust using different CT scanners and acquisition protocols. All the patients in the study had presented with chest pain and associated symptoms that indicated an intermediate risk of coronary artery disease. All patients underwent cardiac CT angiography for anatomical assessment of their coronary arteries and risk stratification for coronary artery disease. The study was carried out in accordance with the recommendations of the South East Research Ethics Committee, Aylesford, Kent, UK, with written informed consent from all subjects in accordance with the Declaration of Helsinki. An example of a CTCA scan is shown in Fig. 1.

### B. Data Pre-Processing

The original Digital Imaging and Communications in Medicine (DICOM) data were pre-processed using 3D Slicer. The brightness of the CTCA image was adjusted by windowing (window center = 40, window width = 400). The image was saved as a NIfTI Data Format. The image size was $512\times512$ pixels. The image was then processed by ImageJ [41]. The pixel intensity was normalised by using linear histogram stretch and then rescaled to between 0 to 255. The final images were converted to 8-bit Portable Network Graphics (PNG) for training, validation and testing. The pre-processing technique is simple and quick to perform.

### C. Semi-Automatic Segmentation

**FIGURE 2.**
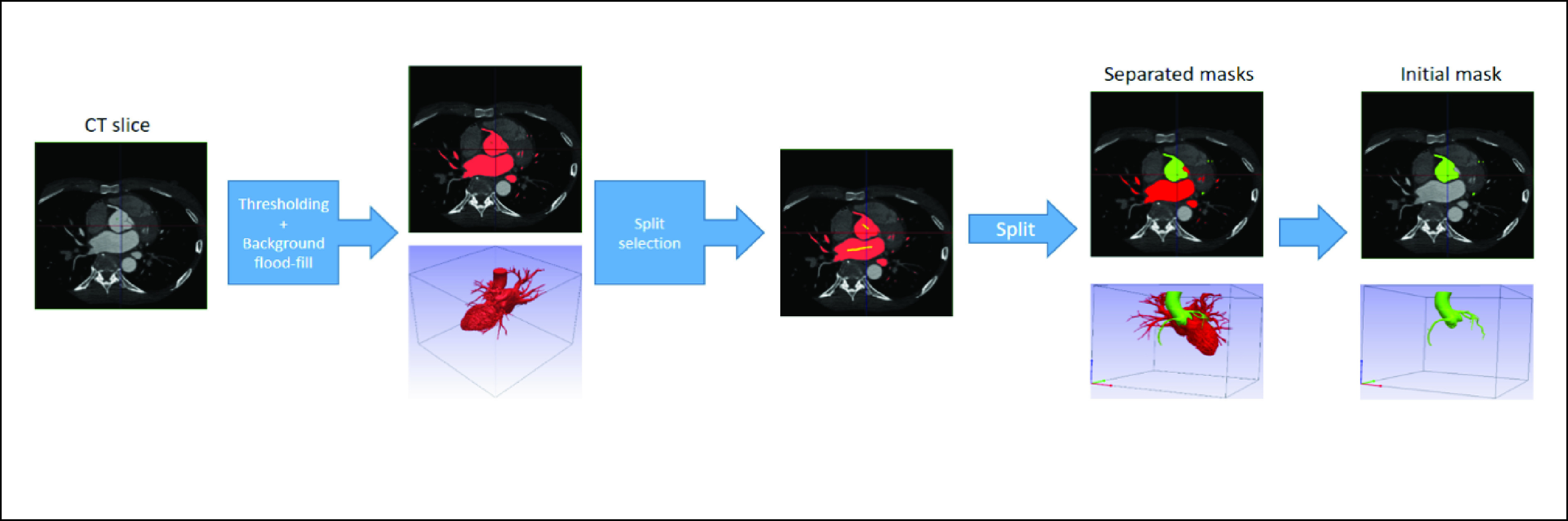
The workflow of initial annotation by using Simpleware-ScanIP.

Initial annotation was performed using Simpleware-ScanIP (Version 2018.12; Synopsys, Inc., Mountain View, USA). The segmentation procedure consisted of thresholding, background flood-fill and split algorithms. Firstly, thresholding was applied such that only regions containing contrast were considered. Secondly, a seed point was placed within the aorta, and the background flood-fill algorithm was able to segment the coronary arteries and cardiac chambers which were connected to the aorta. Lastly, the split algorithm was performed such that the aorta and the coronary arteries were separated from the cardiac chambers. It should be noted that the split operation may be repeated such that all connected chambers are separated. The workflow of these procedures is displayed in Fig. 2.

**FIGURE 3.**
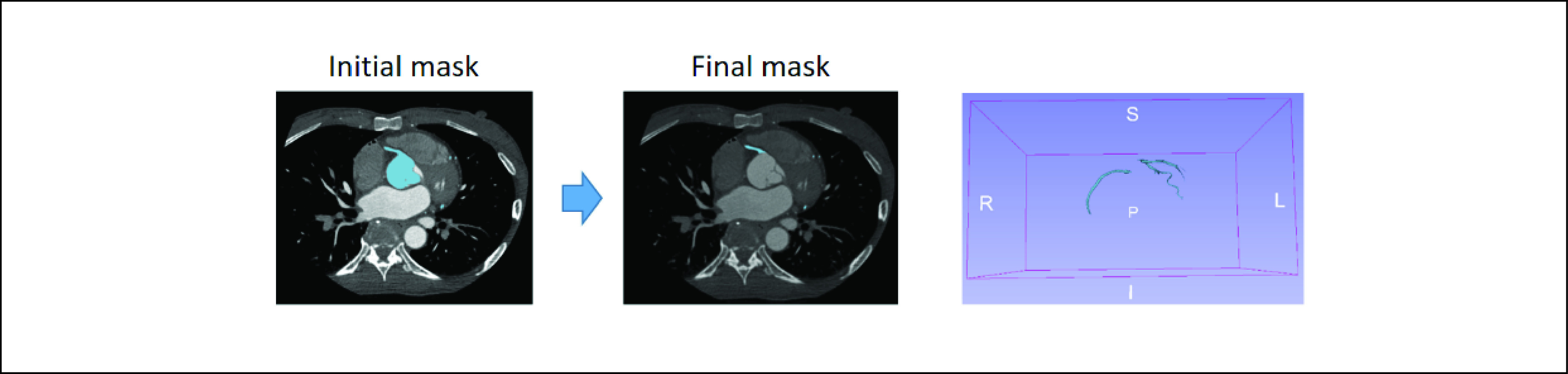
The final coronary artery mask fine-tuned using 3D slicer.

The initial mask contained the ascending aorta (AA), right coronary artery (RCA), left circumflex artery (LCX) and left coronary artery (LCA). The mask was then fine-tuned manually using 3D Slicer [42]. To acquire the mask containing just the coronary arteries, the AA was removed to leave the RCA, LCX and LCA only. An example of initial and final masks is shown in Fig. 3.

### D. Manual Segmentation

The manual segmentation was implemented in Slicer 3D. The annotator highlighted the vessel by identifying the contrast within the CTCA image given an initial mask. The segmented masks were used as the optimum manual (ground-truth) labels.

### E. Segmentation Methods

#### 1) Aorta and Coronary Artery Segmentation

The regions of interest that the current work focusses on are the aorta and coronary arteries. There are two scenarios where these masks are used. (1) The combined mask of the aorta and coronary arteries is useful for blood flow estimation using computational fluid dynamics. Aorta segmentations can produce continuous 3D measurements of aortic size and shape which are objective and allow detailed longitudinal comparisons of subtle changes in aortic morphology for various disease states of the aorta. (2) The mask of coronary arteries alone is useful for cardiologists to assess the degree of stenosis in areas where CAD has developed. The proposed method was therefore evaluated on these two segmented masks.

#### 2) Fully Automatic Segmentation

##### a: Our Proposed Model

**FIGURE 4.**
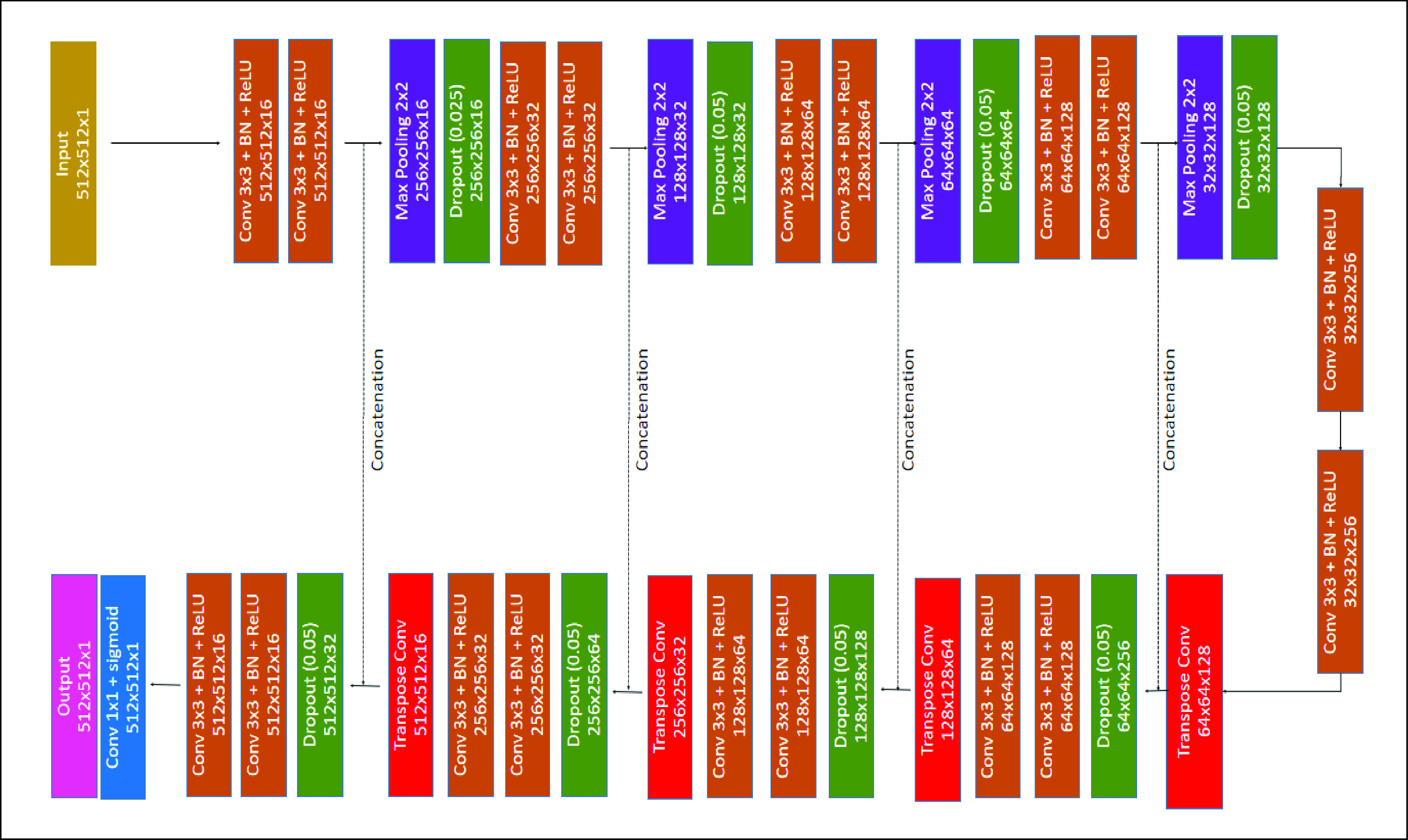
The network architecture of our proposed method.

Our model is based on the 2D Unet [40]. A Unet is a deep convolutional neural network consisting of down-sample and up-sample paths. The first component of the network extracts spatial features and contexts, while the second component localizes the features by using transposed convolutions. A sigmoid function is used for the final background/foreground classification. We have modified the Unet model in two ways: (1) a batch normalization layer is added to the convolution block; (2) a dropout layer is added before each convolution block. The training process suffered from internal covariate shift. The batch normalisation was able to stabilise the training by normalising the inputs for each mini-batch which was performed by computing the mean and standard deviation of each input variable to a layer per mini-batch. The dropout layer was used to reduce overfitting by setting the weights to be zero randomly. This additional implementation improved the stability and performance of the proposed model. The details of our proposed model is shown in Fig. 4.

##### b: Model Training

55 datasets (n = 13 with no coronary disease, n = 42 with coronary disease) were used for training (80%) and validation (20%) The test data set contained 14 datasets (n = 5 with no coronary disease, n = 9 with coronary disease). There were 11677 slices in the training dataset and 2920 slices in the validation dataset. Slice by slice training was adopted. Two models were trained by using the following optimum manual labels: (1) Aorta and coronary arteries (2) coronary arteries only.

##### c: Training Implementation

The proposed models were implemented in Tensorflow (v 2.1.0) and Keras (2.3.1) on Linux (Rocks 7). They were executed on a cluster (Intel Xeon Gold 5118, 2.3GHz) with a Tesla V100-PCIE-32GB GPU. The Adam algorithm was used to optimise the proposed models. The learning rate was initially set to 1e-5. 200 epochs were set for model training. The only hyperparameter optimised in this study was the learning rate. It was initially set as 1e-5. When the DSC had stopped improving after three epochs, the learning rate was reduced by a factor of 0.1 using Keras function – ReduceLROnPlateau. The setting was as follows: factor = 0.1, patience = 3, min_lr = 0.00001. Early stopping was executed when the loss was not reduced across 10 consecutive epochs.

##### d: Loss Function and Performance Evaluation

The combined binary cross entropy (BCE) and dice similarity coefficient (DSC) with equal weights were used as the loss function for deep learning. The segmentation performance was measured by using DSC and IoU metrics which are commonly used to measure the similarity between two segmentations.

##### e: The Segmentation Prediction Implementation

The prediction was performed by using the trained models above. It was implemented on Tensorflow (v 2.1.0) and Keras (v 2.3.1) on Windows 10 and executed on a machine (Intel i9-9960X, 3.1GHz) with a Nvidia Geforce RTX 2700 GPU. The time required for the prediction was also recorded on a per subject/patient basis.

#### 3) Segmentation Performance and Time Evaluation

The accuracy of the segmentation performance of our proposed method was compared with published accuracies of existing 2D and 3D deep learning models. Our model was also compared with the standard Unet++ [43] and its variant that incorporating Xnet [44] with batch normalisation. It should be noted that the standard skip connections in Unet only combine the decoder feature maps with the same scale feature maps from the encoder, this could limit its ability to capture the intermediate features maps at multiscale levels. The redesigned skip connections mechanism in Unet++ could be used to overcome this limitation and hence a better artery segmentation might be obtained.

For the test dataset, the time required for segmentation and the segmentation performance for our method was compared to semi-automatic segmentation methods. The performance of the segmentation was evaluated by using the DSC and IoU metrics. The Mann-Whitney U Test was performed to evaluate whether there was any difference in segmentation time between automatic and semi-automatic approaches. The analyses were implemented on SPSS (IBM SPSS Statistics for Windows, version 25, IBM, Armonk, NY, USA).

## III. Experiments and Results

### A. Learning Curve

**FIGURE 5.**
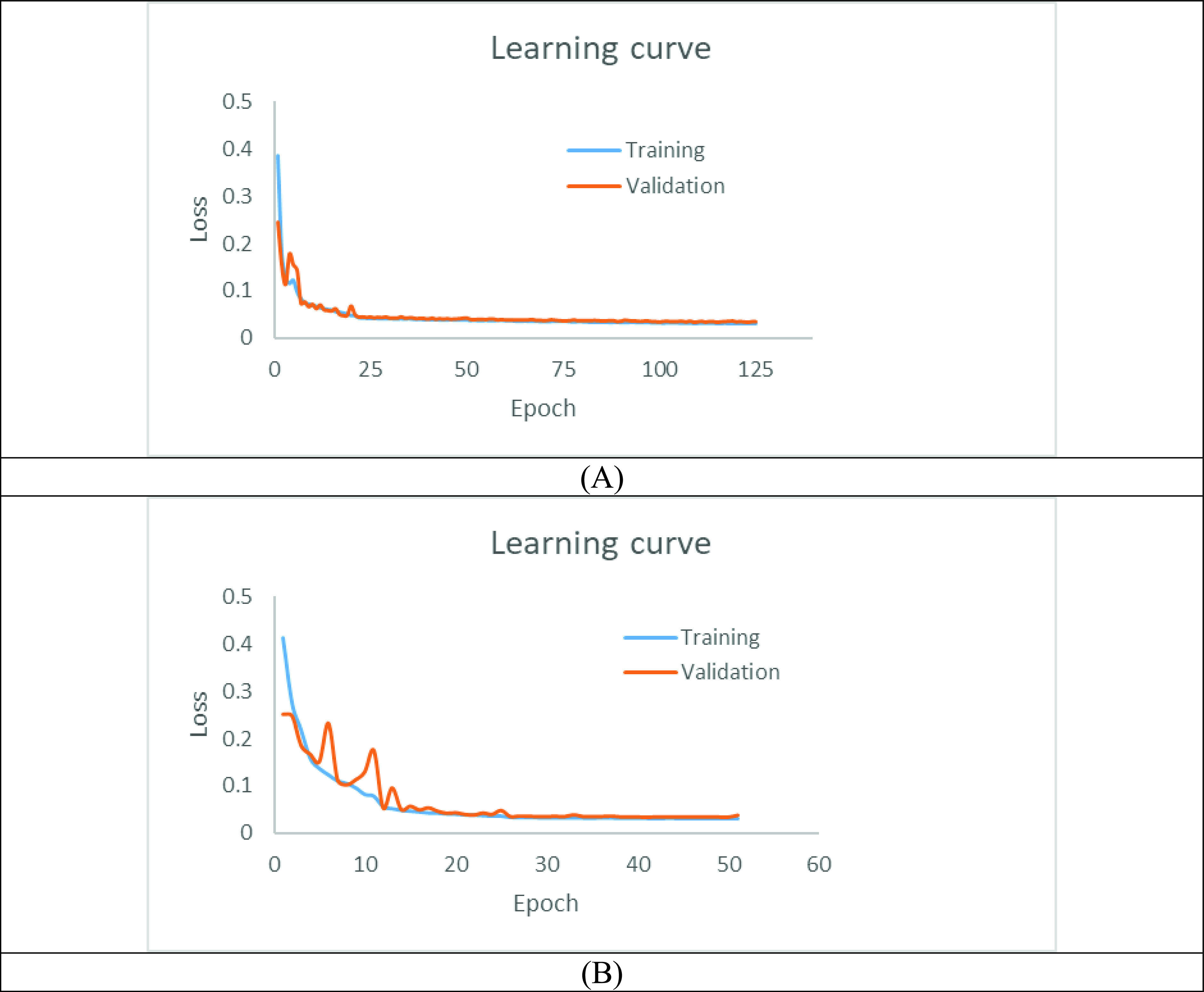
Learning curves: trained with (A) aorta and coronary arteries (B) coronary arteries only.

The learning curves of our model for two scenarios are shown in Fig. 5. No overfitting was found in the training for both scenarios. Some fluctuations of the loss function were found at an early stage of training, though the training became stable later on. This potentially reflects the fact that the training was performed in mini-batches. The training that used the aorta and coronary arteries as ground-truth labels took 125 epochs, while the training using coronary arteries alone as the ground-truth label took only 51 epochs. This indicates that the aorta and coronary arteries have distinct features that took longer to learn in the first scenario.

**FIGURE 6.**
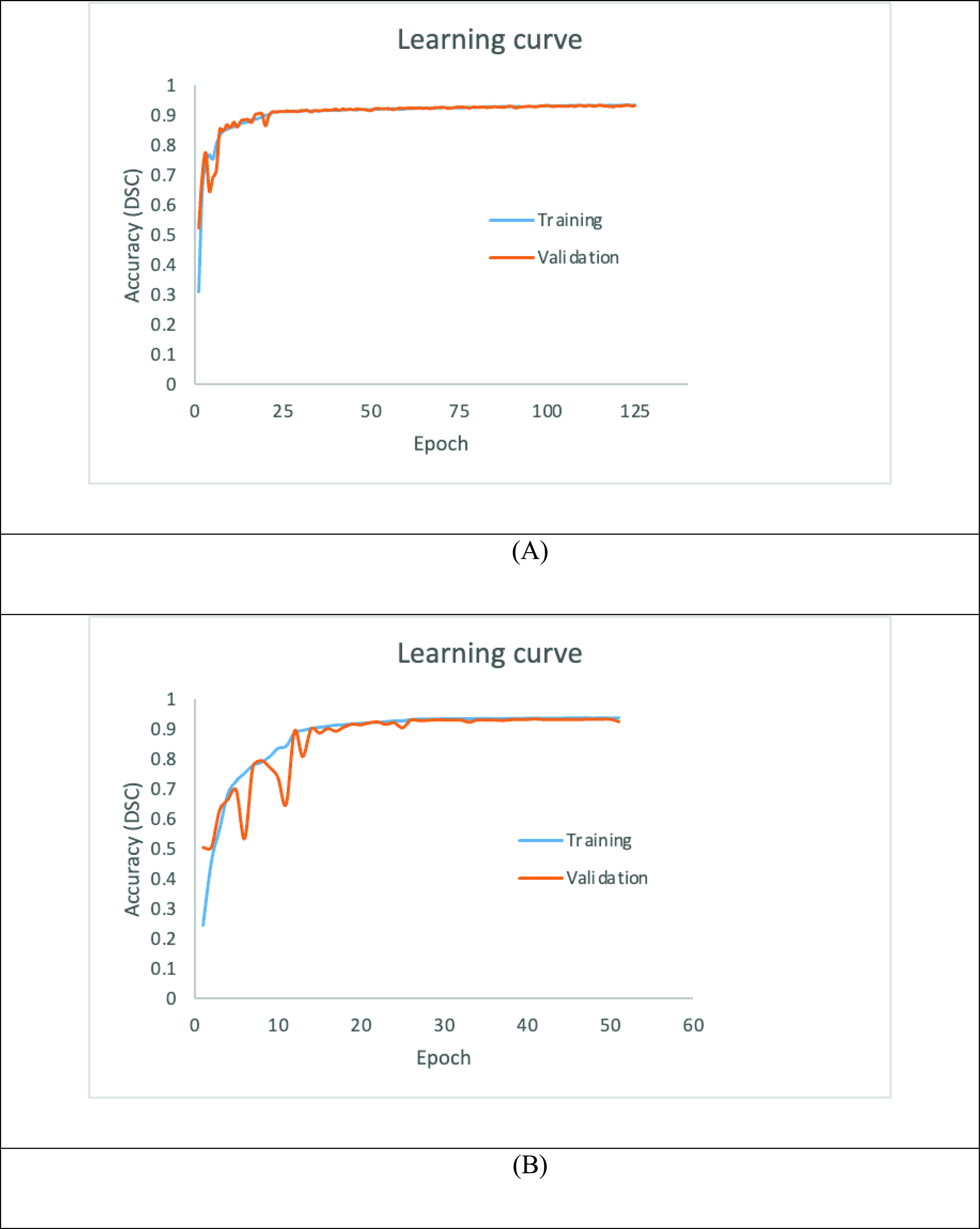
Learning curves – accuracy (DSC): trained with (A) aorta and coronary arteries (B) coronary arteries only.

The accuracy (DSC) during training is shown in Fig. 6. Within the same model, it can be seen that the segmentation performance improves as more features are learnt.

### B. Effect of Network Depth

In general, a deeper network would be able to capture the main/larger diameter artery on the CTCA image, while a shallow network would be able to capture the branch/narrow diameter artery on the CTCA image. For this work, an optimization study was performed (see Table 1). We found that the optimal results (performance on test set) were obtained when using a four-layer (number of layers over which skip connections were used) network.

**TABLE 1:** Effect of Network Depth to Segmentation Performance

	Aorta and coronary artery			Coronary artery only		
DSC (average)	Training	Validation	Test	Training	Validation	Test
1-layer	27.35%	26.95%	24.14%	51.48%	50.62%	48.14%
2-layer	66.80%	67.13%	67.60%	66.46%	68.65%	73.34%
3-layer	86.24%	86.79%	85.36%	87.74%	87.51%	82.63%
4-layer	93.62%	93.33%	91.20%	93.82%	93.41%	88.80%
5-layer	95.06%	94.98%	88.78%	94.63%	94.49%	88.16%
6-layer	95.37%	95.13%	87.81%	94.94%	94.55%	88.22%
7-layer	94.83%	94.99%	88.97%	94.56%	94.39%	87.58%

### C. Segmentation Performance

Table 2 shows the segmentation performance when the aorta and coronary arteries were segmented. The accuracy of our method and Simpleware-ScanIP are 91.20% and 99.40% respectively. The semi-automatic approach performed better than our method when both the aorta and coronary arteries were present in the mask.

**TABLE 2:** Segmentation Performance – Mask Contains Aorta and Coronary Arteries

With aorta	DSC (average)	IoU (average)
Simpleware-ScanIP	99.40%	98.81%
Our method	91.20%	83.82%

The performance for segmentation of the coronary arteries alone is shown in Table 3. The accuracy of our method and Simpleware-ScanIP were 88.80% and 73.22% respectively. Our method performs better than the semi-automatic approach when just the coronary arteries are present in the mask.

**TABLE 3:** Segmentation Performance – Mask Contains Coronary Arteries Only

Without aorta	DSC (average)	IoU (average)
Simpleware-ScanIP	73.22%	57.75%
Our method	88.80%	79.85%

**FIGURE 7.**
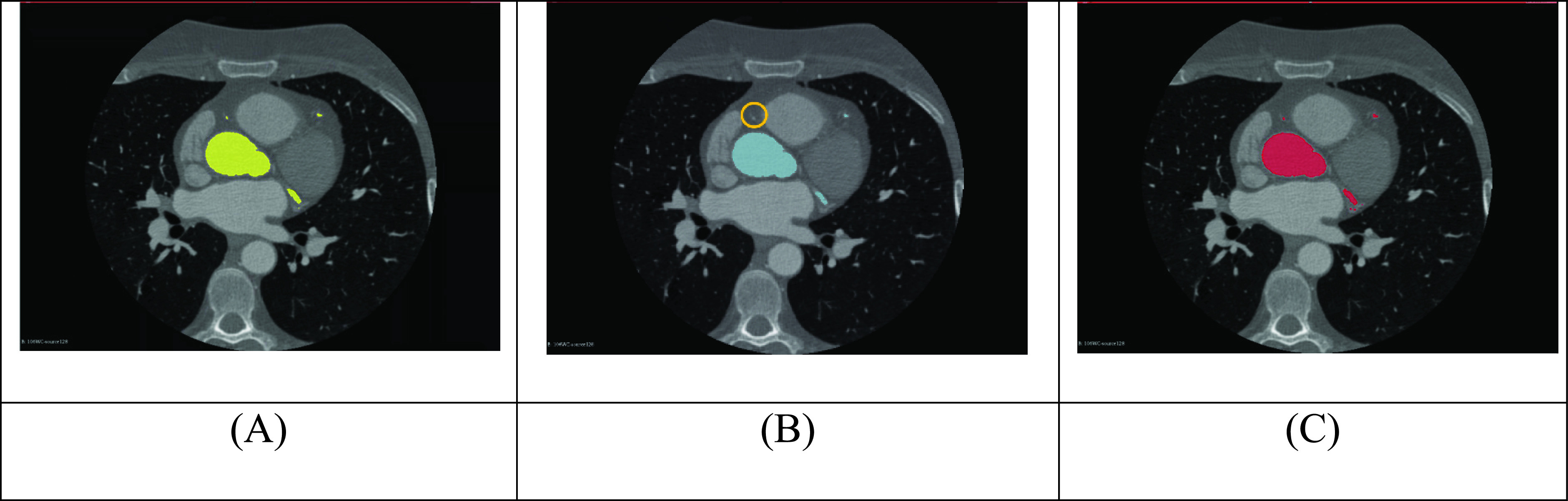
(A) Ground-truth mask (yellow) (B) Mask from Simpleware-ScanIP (blue) with a missing vessel (orange circle) (C) Mask from our model (red).

The results demonstrate that a semi-automatic approach is good at segmenting the aorta. The semi-automatic approach was limited in its ability to segment the coronary arteries, but as the aorta occupied most of the volume of the mask, the overall segmentation accuracy remained high. Our method performed well when attempting to segment the coronary arteries alone. This suggests that our model has the ability to utilise other features (i.e. shape) to recognise the coronary arteries, while the semi-automatic approach relies solely on pixel density. If the contrast within the coronary artery is not bright enough, the semi-automatic approach will miss some segments of the coronary artery (See Fig. 7).

**FIGURE 8.**
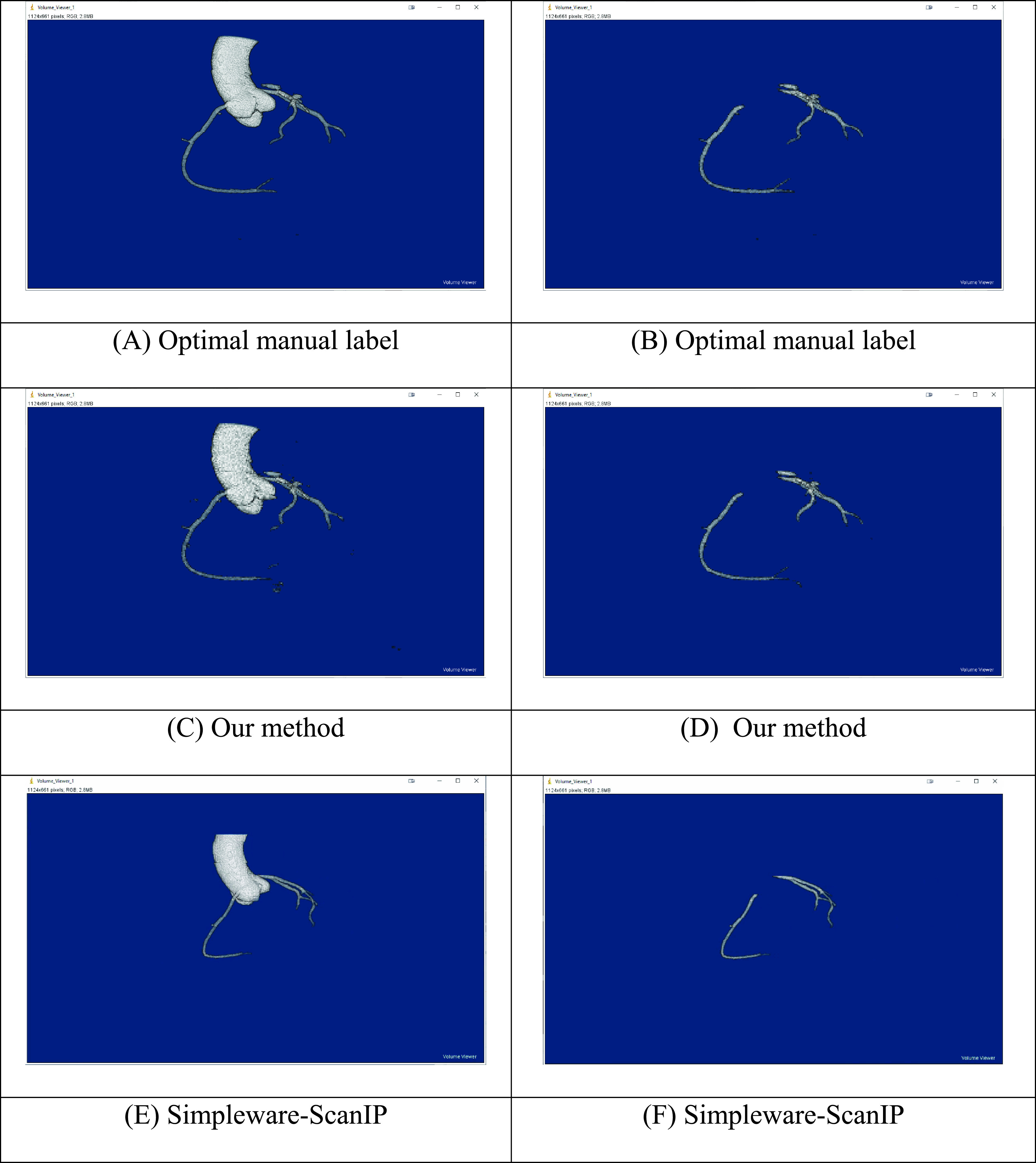
Segmentation results of patient 1. Segmentation of aorta and coronary arteries: (A) Optimal manual label, (C) Our method, (E) Simpleware–ScanIP. Segmentation of coronary arteries only: (B) Optimal manual label, (D) Our method, (F) Simpleware–ScanIP.

**FIGURE 9.**
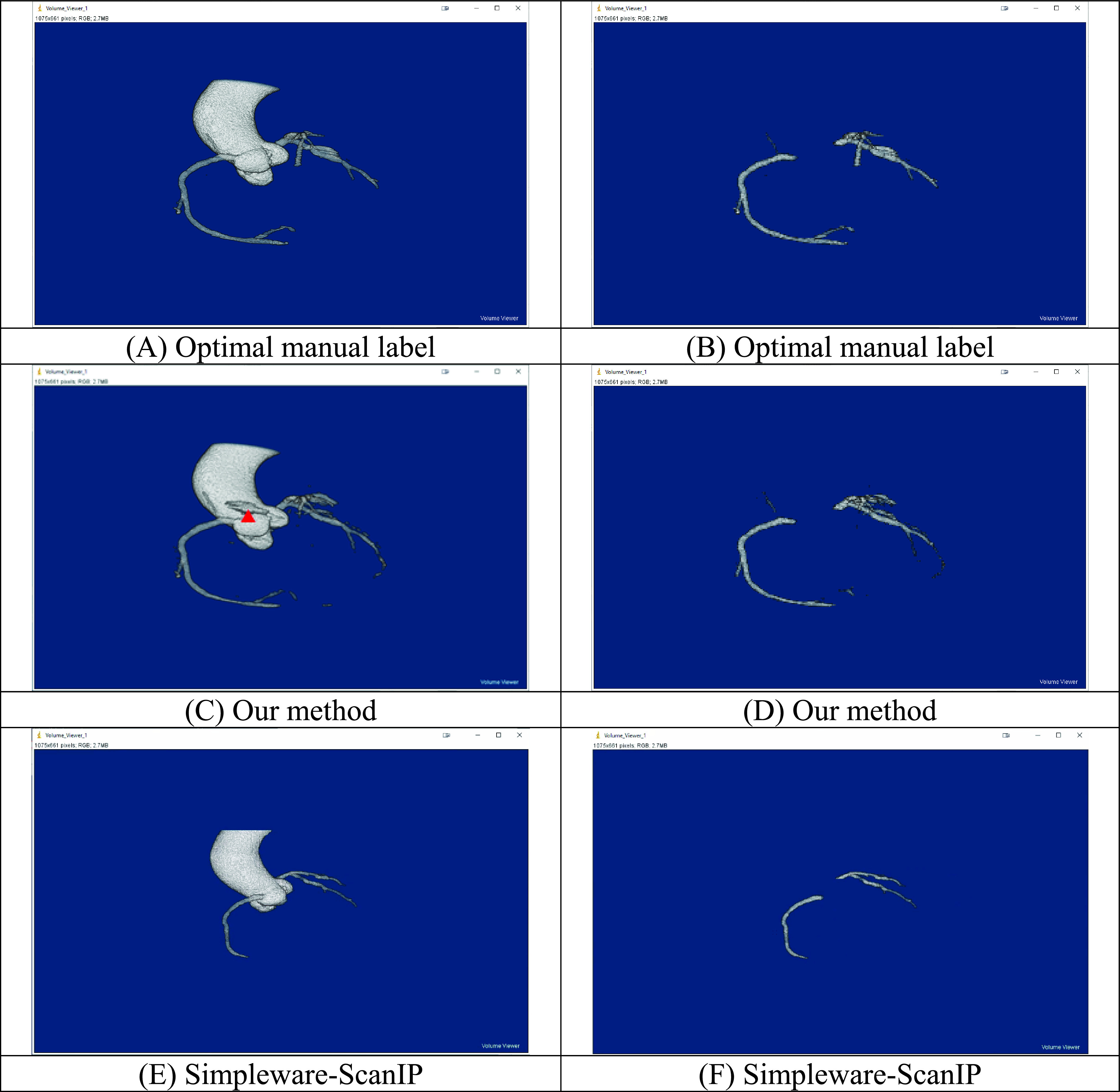
Segmentation results of Patient 2: with aorta and coronary arteries (A) Optimal manual label (C) Our method (E) Simpleware–ScanIP, with coronary arteries only (B) Optimal manual label (D) Our method (F) Simpleware–ScanIP.

The segmentation results of patients 1 and 2 are displayed in Figs. 8 and 9. From Fig. 8, it is clear that our method can segment the aorta and coronary arteries, with a result very close to the optimal manual label. The Simpleware–ScanIP segments the aorta with good accuracy while some segments of the coronary arteries are missing.

For patient 2 (Fig. 9), our method can segment the aorta and coronary arteries well, but the segmentation incorporates an artefact (red arrowhead). When segmenting the coronary arteries alone, some segments of the coronary arteries are missing in the segmentation. As expected, the aorta segmentation is good when using the semi-automatic method, while the segmentation of the coronary arteries is relatively poor. It should be noted that the artefact present in the segmentation of the aorta and coronary arteries using our method can be easily removed by excluding the non-connected components of the mask.

### D. Segmentation Performance Comparison With Unet++

**FIGURE 10.**
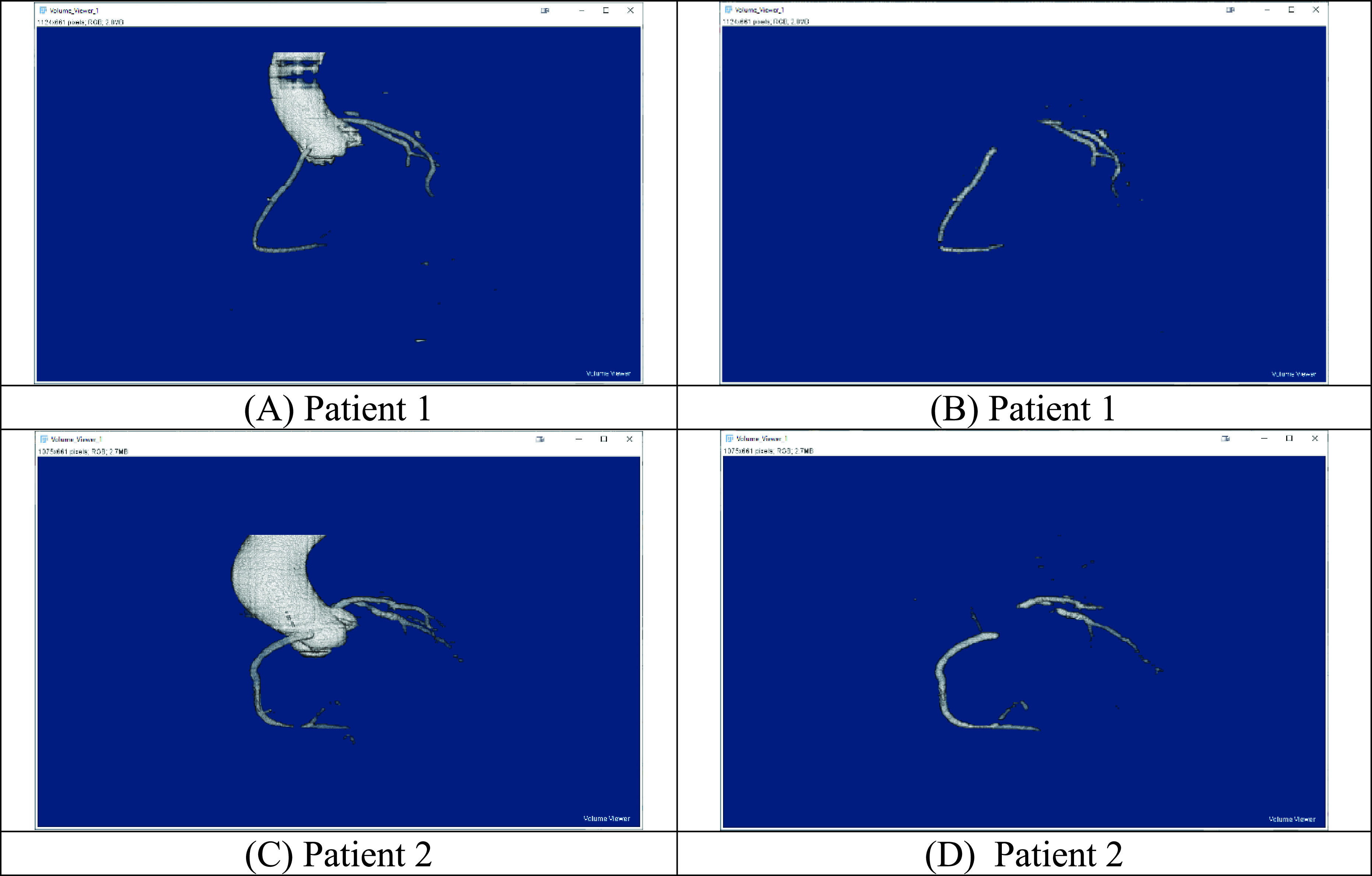
Segmentation results by Unet++ [Xnet/BN]. Segmentation of aorta and coronary arteries: (A) Patient 1, (C) Patient 2. Segmentation of coronary arteries only: (B) Patient 1, (D) Patient 2.

Table 4 shows the segmentation performance of the standard Unet++ and its variant [Xnet with batch normalisation]. From Table 4, the performance of the standard Unet++ is the weakest. This may be due to the fact that batch normalisation has not been adopted to reduce the internal covariate shift which has affected the training significantly. While the Unet++ variant performed much better than standard Unet++, its performance remains slightly worse than our model. This suggests that learning through semantically similar feature maps may not be useful for artery segmentation. The segmentation of the aorta and/or coronary artery using the Unet++ [Xnet/BN] is shown in Fig. 10.

**TABLE 4:** Segmentation Performance of Standard Unet++ and its Variant [Xnet With Batch Normalisation]

	Aorta and coronary artery				Coronary artery only		
DSC (average)	Training	Validation	Test	Training		Validation	Test
Standard Unet++ (L^1^)	27.47%	26.95%	24.14%		51.48%	50.62%	48.14%
Standard Unet++ (L^4^)	27.47%	26.95%	24.14%		51.48%	50.62%	48.14%
Unet++ [Xnet/BN] (L^4^)	85.81%	79.61%	86.27%		86.67%	76.27%	78.11%

### E. Segmentation Time

The segmentation time of our proposed method compared to semi-automatic segmentation is shown in Table 5 (aorta and coronary arteries) and Table 6 (coronary arteries only) respectively. The mask prediction using our method was significantly faster than the Simpleware-ScanIP for both segmentation scenarios (p-value < 0.001), taking less than 4 seconds on average to predict the aorta and/or coronary arteries masks. Additionally, the segmentation time was between 40s and 141s when run on a CPU with multi-cores.

**TABLE 5:** Segmentation Time – Mask Contains Aorta and Coronary Arteries

With aorta	Time (average)	Time (SD)
Simpleware-ScanIP	203.07s	101.69s
Our method (GPU)	3.29s	0.47s
Our method (CPU only – 2 cores, 4 threads)	138.57s	15.74s
Our method (CPU only – 16 cores, 32 threads)	40.93s	4.71s

**TABLE 6:** Segmentation Time – Mask Contains Coronary Arteries Only

Without aorta	Time (average)	Time (SD)
Simpleware-ScanIP	345.00s	113.45s
Our method (GPU)	3.36s	0.50s
Our method (CPU only – 2 cores, 4 threads)	140.07s	16.90s
Our method (CPU only – 16 cores, 32 threads)	40.93s	4.75s

## IV. Discussions

A deep learning model based on a 2D Unet has been developed to segment the aorta and/or coronary arteries on cardiac CTCA images. Two models were trained to segment ROIs in two scenarios - (1) the aorta and coronary arteries (2) coronary arteries only. Our method demonstrates 91.20% and 88.80% DSC accuracy on scenarios 1 and 2 respectively. This suggests that our method can segment the aorta and/or coronary arteries with high accuracy.

Current deep learning approaches require high computational resources, which are difficult to implement in standard hospital networks. Therefore, we developed an alternative deep learning approach (2D Unet) that is feasible for implementation in a hospital network and which importantly maintains good segmentation accuracy.

Compared with a published 3D deep learning model [22] developed to segment the aorta and coronary arteries, which uses a 3D global feature embedded network with active contour loss, the performance of our method was similar (DSC: 91.20% (our method) vs 91.43% ([22])). Our proposed method utilised a smaller number of network parameters resulting in more efficient training and a faster prediction time compared to the published 3D model. Our method requires less GPU memory, which is a common limitation when training and implementing a 3D model. It should also be noted that our method does not require GPUs for deployment, which favours its application in hospital networks where typically only CPUs are available.

The performance of our method was also compared with the 2D RCNN + 2D Unet technique [36] for scenario 2. Our method showed comparable performance (IoU: 79.85% (our method) vs 84.36% ([36])), but does not require implementation of a sequence model to detect the coronary arteries. Implementing an additional sequence model would increase the computational complexity and hence decrease the algorithmic efficiency. It should be noted that the difference in reported accuracy between these methods are likely to relate to the different test sets that were evaluated.

Compared with semi-automatic methods, our model performance is degraded when segmenting the aorta and coronary arteries. However, our model gives improved accuracy when segmenting the coronary arteries alone. The findings highlight the importance of evaluating segmentation performance of large vessels and small vessels separately to reduce the potential bias of segmentation performance metrics. In terms of the prediction time, our proposed model provided the fastest prediction when compared with the semi-automatic method. Though the time difference is statistically significant, it may be negligible from a clinical perspective.

Our study utilised a larger sample size for training and prediction when compared to the published 2D approach. This allows for a more generalizable model and therefore a more reliable prediction. It should be noted that two cases were excluded in our analysis as the Simpleware ScanIP failed to segment the vessels. Our deep learning model however was still able to segment the aorta and coronary arteries adequately.

There are several limitations to this study. The design of the study was retrospective, and accordingly it may have suffered from patient selection bias. The ground-truth labels of the study were obtained by manual annotation and it is possible that the accuracy of the labels were potentially biased due to the annotator’s experience. The performance of our model was compared with existing models using different datasets. The lack of openly available code for other models precluded direct comparisons of models on the same dataset. Although our method can predict the segmented mask with good accuracy, visual inspection of the imaging by experts is still required. Currently, small regions of the proximal coronary artery are occasionally missed when using our models. Further improvements could be made by incorporating an attention gate to our model, which could allow the network to focus more closely on the coronary arteries during training.

## V. Conclusion

Our study demonstrates that a 2D UNET model is able to segment the coronary arteries efficiently and with good accuracy. It has the advantage that it can be deployed within hospital computer networks where GPUs are not available. Our study is a first essential stage of work to develop fully automatic detection and classification systems for coronary artery disease by using computer-based deep learning algorithms. The aims of our future work would be to extend our model for coronary artery disease severity classification which could speed up the patient pathway for referrals with chest pain in A&E departments.
